# Supplementary material for: Necrotic cardiac myocytes skew macrophage polarization towards a classically activated phenotype
Source: PLoS One. 2023 Mar 30;18(3):e0282921. doi: 10.1371/journal.pone.0282921 (PMC10062615; doi:10.1371/journal.pone.0282921)
Supplement: Attachment — Submitted filename: DAMP_Macs Rebuttal (12-22-22).docx [file pone.0282921.s002.docx]

**Comments to the Editor**

We are very appreciative of the careful reading that you gave to the manuscript entitled “Necrotic cardiac myocytes skew macrophage polarization towards a classically activated phenotype” PONE-D-22-27365. We have revised the manuscript in response to your suggestions. For you reference, your original comments have been copied and are shown in bold. The specific responses to your suggestions are given below each comment, along with the appropriate lines in the revised text. We believe that the manuscript is significantly improved based on your suggestions, and hope that you will find the revised manuscript acceptable for publication.

1. **I would like to suggest authors to demonstrate the properties of proteolytic lysate by western blot using cardiomyocyte markers as well as standard Coomassi Blue - or similar staining of the same gel. I am curious whether varying amount of PK or other factors has any  association with macrophage polarization.**

**Author`s Response:** The editor raises and important point which we addressed in a prior study (PMID: 2603708), wherein we developed the methodology to study DAMP biology. As shown in panel A of the supplemental figure S-1, Western blotting of necrotic myocardial extracts (NMC) revealed the present of known DAMPs, including HGMB1 galectin3, S100b, S100A8, S100A9, and IL-1a. To determine whether the effects of the NMCs myocardial extracts were secondary to protein, DNA or RNA we pretreated the necrotic extracts with heat denatured NMCs, and RNase- and DNase-treated NMC supernatants. As shown, in Figure S1B the effects of the NMCs on fibroblast proliferation (BRDU incorporation) were dependent on the concentration of on NMC’s that were used. Moreover, the effects of NMCs were abolished by heat denaturation but were not abolished by treatment with RNAs or DNAs, suggesting that the biological effects of the NMC’s were mediated by proteins and not RNA or DNA.

Supplemental Figure S-1: Effect of necrotic myocardial extract (NMC) supernatants on fibroblast proliferation. A, Representative Western blot analyses of necrotic myocardial extract supernatants for known DAMPs, including HGMB1, galectin3, S100b, S100A8, S100A9, and IL-1a. B, Effect of diluent (PBS), NMCs, denatured NMCs, and RNase- and DNase-treated NMC supernatants on BrdU incorporation in mouse cardiac fibroblasts (n=6 wells/experimental group from 3 independent experiments) From Zhang et al (PMID: 2603708)

**Comments to Reviewer #1**

We are very appreciative of the careful reading that you gave to the manuscript entitled “Necrotic cardiac myocytes skew macrophage polarization towards a classically activated phenotype” PONE-D-22-27365. We have revised the manuscript in response to your suggestions. For you reference, your original comments have been copied and are shown in bold. The specific responses to your suggestions are given below each comment, along with the appropriate lines in the revised text. We believe that the manuscript is significantly improved based on your suggestions, and hope that you will find the revised manuscript acceptable for publication.

**1. The current study did not distinguish the NCE from cardiomyocytes from other cells, so it is unclear whether the current effect is specific to myocardial infarction of universal. The authors should make NCEs from other cells and compare the effects.**

**Author`s Response:** The reviewer raises an important point which we addressed in a prior study (PMID: 2603708), wherein we developed the methodology to study DAMP biology. Supplemental figure S-1A, when we treated fibroblast cultures with necrotic liver extracts there was a concentration-dependent increase in fibroblast proliferation. Figure S-1B shows the biological effects of necrotic myocardial extracts were quantitatively similar to those observed with necrotic liver extracts, suggesting that the biological effects of DAMPs are a property of different cell types and not just confined to cardiac myocytes. Figures S1A and S1B also show that the effects of necrotic liver and myocardial extracts were abolished by heat denaturation of the extracts, but not by DNAse or RNAse, suggesting that the biological effects of DAMPs are mediated by proteins.

**Supplemental Figure S-1**. A, Effect of necrotic liver cells (NLCs) on BrdU incorporation in NIH/3T3 fibroblasts in the presence and absence of heat denaturation and RNAse and DNAse pretreatment. B, Effect of necrotic myocardial cells (NMCs), denatured NMCs, and RNase- and DNase-treated NMC supernatants on BrdU incorporation in mouse cardiac fibroblasts. From Zhang et al (PMID: 2603708)

2. **The current study did not analyze the receptor to mediate the current effect. Different PRR receptor inhibitors should be used to analyze which PRRs are involved.**
   **Author`s Response:** The reviewer raises an important point which we addressed in a very recent study (doi:10.1016/j.jacbts.2022.05.006), wherein we stimulated peritoneal derived CD64+ macrophages with necrotic myocardial extracts (NCEs), LPS (to stimulate the TLR4 receptor) and Pam3CSK5 (to stimulate the TLR2 receptor). As shown in Figure S-2A, treating CD64+ macrophages with LPS and Pam3CSK5 mimicked the effects of necrotic myocardial extracts on the expression of programmed death receptor-1 (PD-1; a marker of macrophage stimulation), suggesting that of necrotic myocardial extracts signaled through TLR4 and TLR2, which is consistent with prior reports in the literature. Figure S-2A shows that the effects of necrotic myocardial extracts on PD-1 expression in CD64+ macrophages could be completely blocked by anti-TLR2 and anti-TLR4 antibodies, further suggesting that of necrotic myocardial extracts signaled through TLR4 and TLR2, consistent with prior reports in the literature. To address the reviewers comments we have added text on lines 316-3019 of the revised manuscript stating that: “Fourth, although we did not identify which receptors were responsible for NCE-induced macrophage skewing in the present study, we have shown recently that necrotic myocardial extracts activate macrophages through both TLR4 and TLR2”

**Supplemental Figure S-2.** Unfractionated peritoneum-derived inflammatory cells (PDICs) were collected following 72-hour culture and the percentage of PD-1+ CD64+ macrophages was evaluated by flow cytometry analysis. A, PDICs were cultured in media alone (control) or necrotic cardiac myocyte cell extracts (NCEs) (10mg/mL), a Toll-like receptor 2 (TLR2) agonist (Pam3CSK4; 200 ng/mL), a TLR4 agonist (lipopolysaccharide [LPS] and the percentage of PD-1 expressing CD64+ macrophages were assessed. B, PDICs were cultured in media alone (control) or in the presence or absence of NCEs (10mg/mL), IgG (4mg/mL), anti-TLR2 antibody (Ab) (4mg/mL), anti-TLR4 Ab (10mg/mL). (From Hayashi et al doi:10.1016/j.jacbts.2022.05.006).

3. **The authors found that protein component is responsible for the classical phenotype polarization. However, more detailed information should be obtained. At least the protein from nucleus, mitochondria and cytosol should be separated to be analyzed in the current polarization study.**

**Author`s Response:** With respect, we do not believe that the suggested experiments will be informative in the present study for several reasons. First, as the reviewer is aware, DAMPs are released abruptly from both the cytosol and nucleus of the cell when a cell undergoes necrotic cell death. Accordingly, the release of the DAMPs from both the nucleus and the cytoplasm into the extracellular environment activates neighboring cells. Second, post-translational modification of DAMPs (e.g. acetylation of HMGB1) can result in DAMPs translocating from the nucleus to the cytoplasm. Thus, the same protein can act as a DAMP if resides in the nucleus or resides in the cytoplasm. We have shown previously that HMBG1, which is both a nuclear and cytosolic protein, is sufficient to activate fibroblasts (PMID: 2603708). Moreover, we have shown that S100A8/9, an intracellular Ca++ binding protein, activates fibroblasts (PMID: 2603708). Lastly, as the reviewer is aware mitochondria contain large amounts of circular DNA (mtDNA) which is inherently pro-inflammatory because it shares many of the same molecular motifs with the bacterial ancestors of energy-producing alpha bacteria that were engulfed by primitive eukaryotic cells 2 billion years ago. The pro-inflammatory effects of proteins that are in the mitochondria are likely overshadowed by the pro-inflammatory effects of mtDNA.

4. **Reactive oxygen special production should be studied.**

**Author`s Response**: In accordance with the reviewers suggestion, we have examined the effects of NCE on reactive oxygen species in isolated peritoneal macrophages. As shown in revised Figure 4D stimulation of macrophage cultures with NCEs led to an increase in ROS production as measured by an increase in the dichlorofluorescin diacetate fluorescence. These findings are discussed in lines 265-269 of the revised manuscript: Given that macrophages produce reactive oxygen species (ROS) during phagocytosis, we asked whether NCE stimulation also provoked increased ROS following NCE stimulation. As shown in Figure 4D stimulation of macrophage cultures with NCE resulted in a significant increase in ROS, consistent with prior studies which have shown that TLR2 and TLR4 engagement are sufficient to by increasing mitochondrial ROS production (20).

5. **The alternative polarization markers are not typical. The authors should use better recognized ones such as IL-10, CD206, Arginase and TGF-beta**

**Author`s Response**: In accordance with the reviewers suggestion, we also examined IL-10 and TGF-beta_1_ mRNA levels which are classical markers of alternative (M2 ) polarization. As shown revised Figure 2, NCE stimulation had no effect on mRNA levels for *Tgfb1 and Il10.* These findings are and discussed on lines 241-243 of the revised manuscript: “NCE treatment significantly (p < 0.001) increased mRNA levels for *Il6*, *Tnf,* and *Nos2*, and significantly (p < 0.05) decreased mRNA levels for *Mgl* and *Retnla,* but had no effect on *Chi3l3,* *Tgfb1 and Il10* mRNA levels.”

6. **The authors should prepare apoptotic bodies to compare the polarization pattern, which also proves the capacity of the macrophages for alternative polarization.**

**Author`s Response:**  We agree with the reviewer that the literature clearly shows that macrophages undergo dramatic molecular and functional changes upon encountering apoptotic bodies, and that efferocytosis of apoptotic bodies skews macrophages towards an anti-inflammatory (i.e. M2) phenotype during the resolution of inflammation. The focus of the present study entitled “Necrotic cardiac myocytes skew macrophage polarization towards a classically activated phenotype” was to study the role of proteins released by necrotic cells in terms of skewing the polarization of macrophages towards a pro-inflammatory (i.e. M1) phenotype

To address the reviewers suggestion, we have added a statement on line 302 of page 14 and lines 320 – 323 of the revised manuscript stating that: “Although the present study was focused on the role of necrosis on macrophage polarization, it should be recognized that efferocytosis of apoptotic cells by macrophages can lead to skewing towards an anti-inflammatory (i.e. M2) phenotype ( reviewed in (PMID: 22566890).

**Comments to Reviewer #2**

We are very appreciative of the careful reading that you gave to the manuscript entitled “Necrotic cardiac myocytes skew macrophage polarization towards a classically activated phenotype” PONE-D-22-27365. We have revised the manuscript in response to your suggestions. For you reference, your original comments have been copied and are shown in bold. The specific responses to your suggestions are given below each comment, along with the appropriate lines in the revised text. We believe that the manuscript is significantly improved based on your suggestions, and hope that you will find the revised manuscript acceptable for publication.

1) **I request the authors to mention that in line 190, the number of differently expressed genes for "LPS only" is 1878. Because from the Venn diagram the total number of differently expressed genes for LPS is 1878+77+123+1205 which is 3283. I request the authors to update the text accordingly in lines 190-192 and 195-196.**

**Author`s Response:** We apologize in advance for any confusion our description of the differentially expressed genes in Figure 1A may have created. Our numbers were referring to “unique” genes that were expressed for each stimulus. To make this clearer we have, amended the text on lines 218-220 to read: “As shown in **Figure 1A,** after 8 hours of stimulation the number of unique differentially expressed genes (fold-change ≥ 2, FDR <0.01) was greatest for LPS (1878 DEGs) > NCE (339 DEGs) > IL-4 (138 DEGs).”

2) **I request the authors to write the magnitude of each bar in figure 1B because with the current y-axis scale for NCE and IL-4 panel, and NCE and LPS+IL-4 panel, it is very difficult to know the height of these bars.**

**Author`s Response:** We apologize in advance for any confusion if the Venn diagrams are not clear. The number of genes (and hence the height of the bars) for NCE and IL-4 are show by the overlap of the purple (NCE) and green (IL-4) circles in Figure 1A (8 hrs = 28, 24 hrs = 26; 72 hrs = 172). The number of genes (and hence the height of the bars) for NCE and LPS + IL-4 as shown by the overlap of the pink (LPS) + green (IL-4) and the purple (NCE) circles in Figure 1A 8 (hrs = 123, 24 hrs = 144; 72 hrs = 556).

3) **In figure 1C, can the authors provide some example genes for each of the ten conditions?**

**Author`s Response:** We have now included a supplemental file with a list of the genes that were up and down regulated following stimulation with NCEs or LPS. There are tabs in the excel spread sheet with gene lists for each of the biological themes in Figure 1C.

We have added text on lines 234-235 to indicate that: (see Supplemental File S-1 for a list of differentially expressed genes that were up and down regulated following stimulation with NCEs or LPS)

4) *It will be helpful if the authors can comment on why they did not see any changes in the* *Chi3l3 mRNA levels in figure 2.*

**Author`s Response:** We used a panel of different mRNAs to assess macrophage polarization. Although there was a numeric increase in *Chi3l3* mRNA levels in the NCE stimulated macrophages, this difference was not significant statistically because of the variability in mRNA expression levels, which was greater for *Chi3l3* than for the other genes in our panel.

5) **It is not clear how the information presented in figure 3 is novel. I request the authors to mention some examples from literature where DNA, RNA, and proteins are shown to be involved in macrophage polarization.**

**Author`s Response:** We apologize in advance for any confusion that the experiments in Figure 3 may have created. Prior studies have shown that proteins (PMID: **27632996**), mitochondrial DNA (PMID: **26067258)**, and extracellular RNA (exRNA), including microRNAs and long non-coding RNAs (reviewed in PMID: **33392206) released by necrotic cells** have been implicated in tissue inflammation and macrophage polarization. The purpose of the studies in Figure 3 was to determine whether the necrotic cellular extracts from cardiac myocytes were secondary to proteins, DNA or RNA. The data in figure 3 show that macrophage polarization by the NCEs was abolished by proteinase K, suggesting that proteins in the NCEs were responsible for macrophage polarization.  **6) I request the authors to label the panels in figure 4. Currently, it does not say	which panel is A, B, and C.**

**Author`s Response:** The panels in the original Figure 4 were labeled as A,B, and C. We have increased the font size and bolded the letter so that these labels to make them more apparent.

**7) In line 250 of the discussion section, the authors mention that necrotic myocyte	extracts contain proteins. Can the authors make some predictions on what	proteins might be playing a role in this mechanism?**

**Author`s Response:** We have previously shown through immunodepletion studies that HMGB1 is an important DAMP in terms of fibroblast activation (PMID: 2603708). We discuss HMGB1 as a potential protein DAMP that is enriched in cardiac extracts on lines of 294-297 of page 14 of the revised manuscript.

**8) In the figure legends why did the authors say S1 Fig 1? This is not a	supplementary figure in the manuscript. Hence, I request the authors to just say	Fig. 1. I also request the authors to make the same correction for the legends of	Fig. 2-4.**

**Author`s Response:** We cannot locate “S1 Figure 1” in the figure legends. Can the reviewer please indicate the lines for this typo and we will correct it

**9) In figure 4, I request the authors to mention what each color represents in the	figure caption.**

**Author`s Response:** We have revised the figure legend on Figure 4 on lines 447-449 to read: “Representative confocal images of uptake of fluorescent beads (green dots) by cultured CD64+ peritoneal macrophages (red) stimulated with diluent, NCEs, LPS or IL-4. DAPI (blue) was used to stain the nuclei.”

**10) There is a typo in line 63 of page 3. I think the authors meant completely instead of complexly.**

**Author`s Response:** Thank you! We have corrected this typo.
